# Systems biology reveals uncoupling beyond UCP1 in human white fat-derived beige adipocytes

**DOI:** 10.1038/s41540-017-0027-y

**Published:** 2017-10-03

**Authors:** Elin Nyman, Stefano Bartesaghi, Rebecka Melin Rydfalk, Sandra Eng, Charlotte Pollard, Peter Gennemark, Xiao-Rong Peng, Gunnar Cedersund

**Affiliations:** 1 Cardiovascular and Metabolic Diseases IMED Biotech Unit, AstraZeneca R&D, 431 83 Gothenburg, Sweden; 2 Department of Biomedical Engineering, Linköping University, 581 85 Linköping, Sweden; 3 Advanced Drug Delivery, Pharmaceutical Science IMED Biotech Unit, AstraZeneca R&D, 431 83 Gothenburg, Sweden; 4 Department of Clinical and Experimental Medicine, Linköping University, 581 85 Linköping, Sweden

## Abstract

Pharmaceutical induction of metabolically active beige adipocytes in the normally energy storing white adipose tissue has potential to reduce obesity. Mitochondrial uncoupling in beige adipocytes, as in brown adipocytes, has been reported to occur via the uncoupling protein 1 (UCP1). However, several previous in vitro characterizations of human beige adipocytes have only measured UCP1 mRNA fold increase, and assumed a direct correlation with metabolic activity. Here, we provide an example of pharmaceutical induction of beige adipocytes, where increased mRNA levels of UCP1 are not translated into increased protein levels, and perform a thorough analysis of this example. We incorporate mRNA and protein levels of UCP1, time-resolved mitochondrial characterizations, and numerous perturbations, and analyze all data with a new fit-for-purpose mathematical model. The systematic analysis challenges the seemingly obvious experimental conclusion, i.e., that UCP1 is not active in the induced cells, and shows that hypothesis testing with iterative modeling and experimental work is needed to sort out the role of UCP1. The analyses demonstrate, for the first time, that the uncoupling capability of human beige adipocytes can be obtained without UCP1 activity. This finding thus opens the door to a new direction in drug discovery that targets obesity and its associated comorbidities. Furthermore, the analysis advances our understanding of how to evaluate UCP1-independent thermogenesis in human beige adipocytes.

## Increased energy consumption explored with mathematical modeling

Beige fat cells induced from the white fat tissue are functionally close to brown fat cells, with increased metabolic activity and potential to increase whole-body energy consumption. In brown fat cells, the high activity is caused by a mitochondrial protein that leaks protons, but the mechanisms for beige fat cells are less understood. The teams of Xiao-Rong Peng, AstraZeneca, Sweden, and Gunnar Cedersund, Linköping University, Sweden, have therefore integrated experiments and biological hypotheses with mathematical models to study metabolic activity in beige fat cells. The researchers find that beige fat cells can be stimulated to consume energy with a mechanism dependent on fatty acids that do not utilize the same leakage protein as brown fat cells. Once characterized, this mechanism and corresponding mathematical models may help identify drug targets to treat obesity.

## Introduction

Obesity and its associated comorbidities are rapidly expanding throughout the developed world. Despite considerable effort to decrease obesity and improve patient outcome little progress has been made, indicating the need for novel treatment options. In the last few years, there has been an increasing hope that such a cure—or at least that part of the solution—can be found through increasing brown adipose tissue (BAT) mass or activity, or both.^1^ The reason for these hopes is that cells in BAT are orders of magnitude more metabolically active than cells in the more prevalent white adipose tissue (WAT). At the heart of this metabolic activity lies the uncoupling protein 1 (UCP1), a mitochondrial inner membrane carrier found in BAT, but not in WAT. UCP1 dissipates the proton motive force (Δ*p*) which is built up by the electron transport chain (ETC), and which normally is used to produce ATP. This increased dissipation results in an increased oxygen consumption rate (OCR), referred to as uncoupled respiration, and increased thermogenesis. Such an increased metabolic activity can also be obtained by converting WAT progenitor cells into brown-like adipocytes (beige/brite adipocytes).

Beige adipocytes, i.e., brown-like adipocytes in WAT, can be induced in rodents by a number of known factors: cold exposure, treatment with thiazolidinediones (peroxisome proliferator-activated receptor gamma [PPAR-γ] agonists) or treatment with β-adrenergic agonists.^2–5^ Pharmacological agents such as rosiglitazone (Rosi; PPAR-γ agonist), triiodothyronine (T3),^6^ bone morphogenic protein 7,^7^ and cardiac natriuretic peptides^8^ have all been reported to induce beige adipocytes also in human cells. While there is no doubt that UCP1 represents the primary mechanism for thermogenesis of brown and beige fat in rodents,^9^ complementary ways to increase energy expenditure independently of UCP1 remains an unexplored field. There are some indications of alternative uncoupling mechanisms that go beyond UCP1: UCP1 knockdown animals can be acclimated to cold temperatures^10^ and are thermogenic responders to adrenergic stimulation.^11^ Such alternative mechanisms may involve mitochondrial carrier proteins such as adenine nucleotide translocator^12^ and permeability transition pore (PTP),^13^ as well as a creatine-driven substrate cycle^14,15^ or the secreted peptidase M20 domain containing 1 enzyme.^16^ Although the ability to induce such alternative mechanisms in human beige cells remains an unexplored field, it would not only imply a step forward in basic biology, but would also open for a new family of drug targets, which could lead us towards new treatment strategies for obesity.

**Fig. 1:**
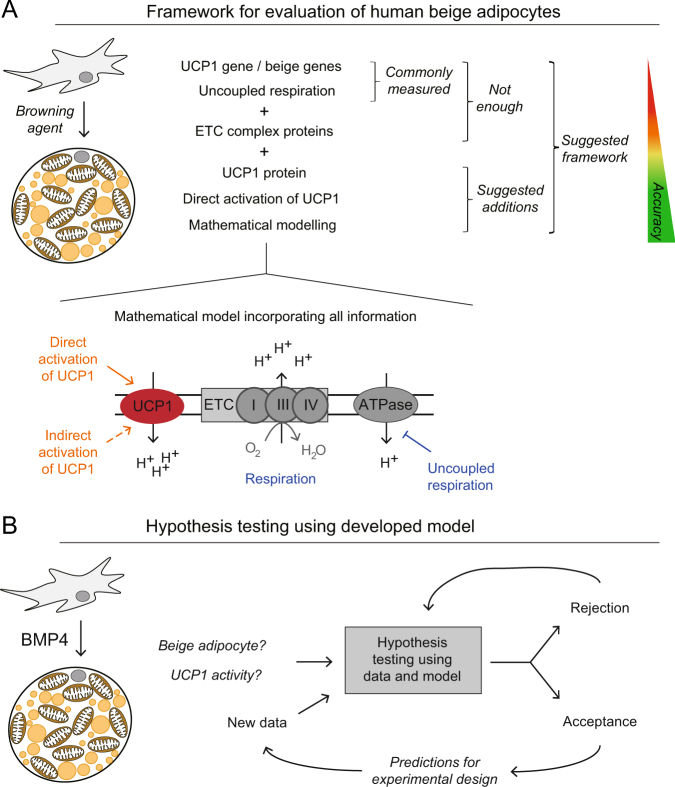
Schematic representation of the framework for evaluation of mechanisms of proton leak in human beige adipocytes. **a** Commonly measured variables like UCP1 mRNA levels are insufficient to properly characterize the bioenergetics system. We show that measurements of UCP1 protein levels and functional responses are required, and mathematical modeling is important to correctly integrate and interpret the data. **b** Hypothesis testing with mathematical modeling is an iterative approach used to reject implausible explanations and thereby advance the understanding. Herein, we have used hypothesis testing to unravel an increased uncoupled respiration in BMP4-treated cells. *UCP1* uncoupling protein 1, *ETC* electron transport chain, *BMP4* bone morphogenic protein 4

In the search for such alternative mechanisms of browning, bone morphogenic protein 4 (BMP4) comes up as an interesting agent.^17^ BMP4 treatments have led to characteristic properties of browning,^18^ but data point in several directions: while BMP4 promotes white adipocyte differentiation^19^ and can reduce recruitment and function of genuine brown adipocytes in rodents,^20^ forced BMP4 expression in WAT of rodents mediates browning and consequent increase in energy expenditure.^17^ Recently, it has also been reported that BMP4 induces a beige expression phenotype in primary human WAT progenitor cells.^21,22^ However, none of these human studies have monitored the level of UCP1 protein or measured its activation. Thus, to fully understand the role of BMP4 in browning, we need a more comprehensive approach, involving a wider array of measurements that also provides a sufficient insight in the metabolic functionality (Fig. 1a). Regarding the induction of beige cells in general, the field is increasingly calling for such more comprehensive approaches to better determine not only if, but in what aspects, a treated cell can be defined as beige.^23,24^

Naturally, more comprehensive data require a more advanced data analysis. Previous research has shown that a correct data analysis in many cases only can be done using mathematical modeling.^25,26^ Several mathematical models for adipocytes have been developed in the past, e.g., models that describe the regulation of the number of adipocytes,^27^ and the intracellular insulin resistance seen in type 2 diabetes.^28–30^ However, none of these models describes brown/beige adipocytes. Models for mitochondrial oxidative phosphorylation have also been reported.^31–33^ These models are based on multiple detailed measurements on isolated mitochondria from cardiac and/or skeletal muscle cells and have been related to clinically measured parameters. However, the same level of detail in data and models do not exist for mitochondria isolated from adipocytes (neither white nor brown/beige adipocytes). Therefore, we see a need for a new generation of models for systematic analyses of metabolic functionality in beige adipocytes.

Herein, we provide a new framework for such analyses (Fig. 1a), which is based on iterations between experiments and modeling, using hypothesis testing and model-based experiment design (Fig. 1b). This framework combines measurements of UCP1 at both mRNA and protein levels with functional and metabolic assessments. We analyze the data using a fit-for-purpose mathematical model for mitochondrial dynamics. The results show that BMP4 induces a state of the cell that shares the metabolic and morphological profile of beige adipocytes, but using another mechanism than UCP1 for proton leak. These insights thus open the door to a new field of research regarding treatments of obesity and its associated comorbidities, and the new framework will be useful for a correct guidance of not only that research, but for corresponding metabolic characterizations of other cell types, such as heart and muscle cells.

## Results

### BMP4 triggers a beige differentiation program in human white fat-derived adipocytes

**Fig. 2:**
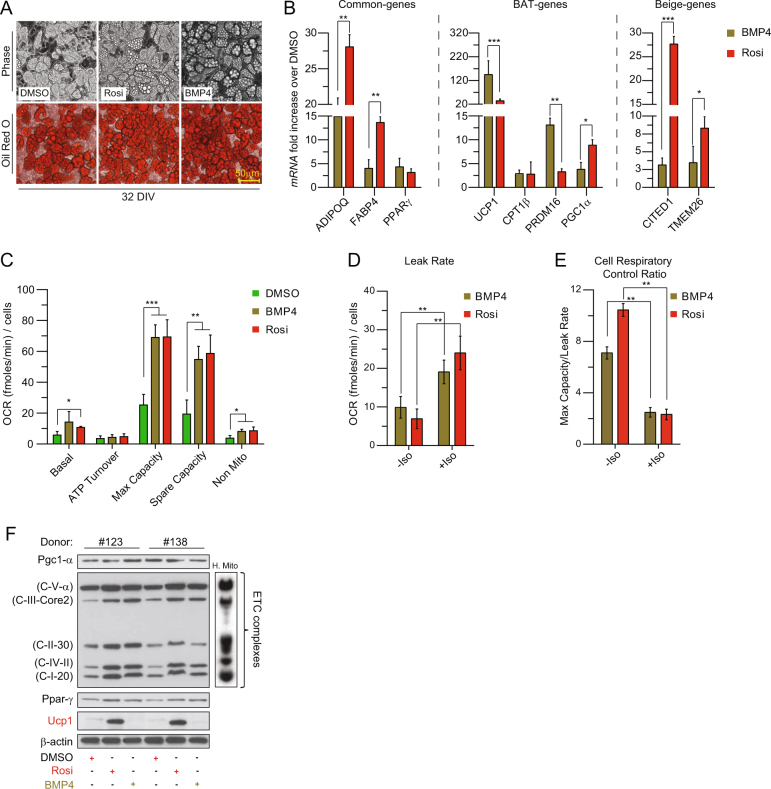
BMP4-treated hASCs undergo beige adipocyte differentiation in vitro and display uncoupled respiration. **a** Oil-Red-O staining of triglycerides in adipocytes derived from DMSO-, Rosi-, or BMP4-treated hASCs. **b** RT-qPCR for the expression levels of genes common to WAT and BAT (left panel), BAT-selective genes (middle panel), beige-selective genes (right panel). **c** OCR for mitochondrial and non-mitochondrial parameters. **d** Oligomycin-insensitive OCR (leak rate, as an index of uncoupled respiration) in in Rosi- and BMP4-treated cells under basal (-) or isoproterenol-stimulated (Iso) conditions. **e** Cell respiratory control ratio (cRCR, as max capacity/leak rate) in Rosi-treated and BMP4-treated cells under basal (-) or isoproterenol-stimulated (Iso) conditions. **f** Representative western-blot analysis (*n* = 2 subjects) of ETC complexes, Pgc1-α, PPAR-γ, UCP1 and β-actin (loading control) in DMSO-treated, Rosi-treated, and BMP4-treated cells. Human mitochondria (H. mito.) heart extract is the loading control for ETC complexes. Data in panels **b**, **c** and **e** are represented as mean ± SEM. * *p* < 0.05, ** *p* < 0.01, *** *p* < 0.001, Students’ *t*-test. *BMP4* bone morphogenic protein 4, *Rosi* rosiglitazone, *Iso* isoproterenol, *UCP1* uncoupling protein 1, *ETC* electron transport chain. See also Supplementary Fig. S2

To assess the effect of BMP4 on differentiation, we cultured human adipose-derived stromal/progenitors cells (hASCs) under adipogenic condition with or without addition of Rosi or the newly-proposed browning factor BMP4^17,21^ during a 32-day differentiation protocol as previously reported.^34^ Oil-Red-O staining confirmed that all treated cultures underwent conversion into lipid droplet-containing adipocytes (Fig. 2a). Gene-expression analysis revealed that both Rosi and BMP4 increased the expression levels of PPAR-γ and its target genes *ADIPOQ* and *FABP4* (Fig. 2b, Common genes). For *ADIPOQ* and *FABP4*, the increase was larger for Rosi than for BMP4 (*p* < 0.01). Rosi-treatment led to ≈150-fold increase in UCP1 (Fig. 2b, BAT genes), which was resembled by increased expression of other brown fat selective-genes such as PRDM16, CPT1β and PGC1α (Fig. 2b, BAT genes). In accordance with reported literature,^21^ BMP4 not only stimulated the expression of common adipose genes, but also activated brown fat-specific genes (UCP1, CPT1β, PRDM16 and PGC1α; Fig. 2b, BAT genes). Comparing Rosi and BMP4 treatment, BMP4 gave a larger increase in *UCP1* (*p* < 0.001) and *PRDM16* (*p* < 0.01) while Rosi gave a larger increase in *PGC1α* (*p* < 0.05). We found that both Rosi- and BMP4-treated human adipose-derived stem cells (hASCs) also upregulated the beige-specific markers CITED1 and TMEM26 (Fig. 2b, Beige genes), Rosi more pronounced than BMP4 (*p* < 0.001 for *CITED1* and *p* < 0.05 for *TMEM26*), while the levels of two other brown fat-specific genes (ZIC1 and EVA1) were unchanged compared to untreated control (data not shown).

Thermogenesis in classic brown fat cells is activated through the β-adrenergic signaling cascade,^35^ and characterized by an acute increase in the rate of proton leak (uncoupled respiration). To assess the potential for respiratory uncoupling in hASC-derived beige adipocytes, we measured cellular respiration (OCR) using the Seahorse instrument (Seahorse Bioscience). The full traces are shown in Supplementary Fig. S2. Detailed analysis showed that Rosi-treatment and BMP4-treatment have comparable levels of basal respiration, ATP turnover, maximum capacity, spare capacity, and non-mitochondrial respiration (Fig. 2c). To dissect the thermogenesis function of hASCs-derived beige adipocytes, we monitored the oligomycin-insensitive OCR (leak rate, as an index of uncoupled respiration) in Rosi-treatment and BMP4-treated cells under basal (-) or isoproterenol-stimulated (Iso) conditions (Fig. 2d). The cell respiratory control ratio (cRCR; defined as Max capacity/leak rate)—analogous to the (uncoupled) respiratory control ratio of isolated mitochondria—was significantly decreased in both BMP4-treated and Rosi-treated cells after Iso stimulation (Fig. 2e), thus supporting the genuine status of the observed uncoupled OCR. In line with these results, the large increase in UCP1 mRNA levels in Rosi-treated cells was reflected at the protein level. Indeed, while UCP1 protein was undetectable in control white cells, it was abundant in Rosi-treated cells (Fig. 2f). Cultures displaying a dramatic increase in the protein levels of UCP1 also displayed a significant increase of representative subunits of the ETC (Fig. 2f). Unexpectedly, however, although the BMP4-treated cells expressed high levels of ETC proteins, UCP1 protein was undetectable (Fig. 2f).

Altogether, these data suggest that both Rosi and BMP4 causes uncoupling, and that BMP4 does so without a corresponding increase in UCP1 protein level.

### Thermogenic activity of UCP1 in human beige adipocytes

**Fig. 3:**
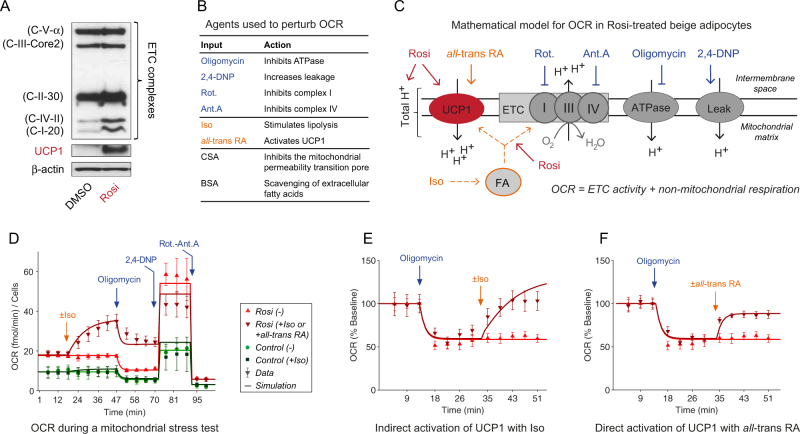
Model development, analysis, and experimental validation of thermogenic activity of UCP1.** a** Representative Western-blot analysis of ETC complexes, UCP1 and β-actin (loading control) in DMSO-treated and Rosi-treated cells. **b** List of agents used to perturb OCR. **c** Mathematical model for OCR in Rosi-treated beige adipocytes. **d** Representative OCR data (markers) and model simulation (lines) in Rosi-treated beige adipocytes (red) compared to control white adipocytes (green) for a mitochondrial stress test. **e**–**f** Data (markers) and model simulation (lines) of uncoupled (oligomycin-inhibited) OCR upon Iso (E) and all-*trans* RA (F) stimulation in Rosi-treated beige adipocytes. Data in panels **d**–**f** are from Rosi-treated cells represented as mean ± SEM. *OCR* oxygen consumption rate, *ETC* electron transport chain, *Rosi* Rosiglitazone, *2,4-DNP* 2,4-Dinitrophenol, *Rot.* Rotenone, *Ant.A* Antimycin A, Iso Isoproterenol, *all-trans* *RA* all-*trans* retinoic acid, *CSA* cyclosporine A, *BSA* bovine serum albumin. See also Supplementary Figs. S1, S3, S4

To unambiguously examine the requirement of UCP1 in the thermogenic activity of derived beige adipocytes, we combined a comprehensive bioenergetics analysis with mathematical modeling using ordinary differential equations (Fig. 1 and Supplementary Fig. S1). In an initial analysis, we focused on the OCR related to Rosi treatment, i.e., OCR in adipocytes that are UCP1 positive (Fig. 3a, Supplemental Experimental Procedures). The level of UCP1 protein was implemented as a model variable to account for the increased uncoupling in Rosi-treated cells (Fig. 3c, red arrow). Further, model parameters for Rosi-induced increased total mitochondria (and thus H^+^ ions) and increased free fatty-acid content were also implemented (Fig. 3c, red arrows). With these parameters representing the difference between control white and Rosi-treated beige adipocytes, we generated a mathematical model for OCR during a mitochondrial stress test (Fig. 3c and Supplementary Fig. S1). The model was fit-for-purpose in the sense that we included only mechanisms needed to simulate the data in this study—not all known/hypothesized mechanisms of mitochondrial dynamics. Therefore, the model could be kept small (~20 parameters). The mitochondrial stress test is a series of mitochondrial perturbations using oligomycin (ATPase inhibitor), rotenone and antimycin-A (complex I and III inhibitors), and DNP-2,4 (uncoupler) (Fig. 3b). The model was designed to be able to simulate these perturbations and also to simulate the response to Iso, which gives an increase in fatty acids that leads to an indirect activation of UCP1 and increased substrate availability, and to all-*trans* retinoic acid (all-*trans* RA, fatty acid-like metabolite), as a direct activator of UCP1^36,37^ (Fig. 3b and c). To parametrize the model, we generated dynamic OCR data in control white and Rosi-treated adipocytes (Fig. 3d). Stimulation with Iso resulted in a 2-fold increase in the oxidation rate of Rosi-treated cells but had no impact on control cells (Fig. 3d). Further, the Rosi-treated cells displayed a significant increase in oligomycin-insensitive (uncoupled) OCR.

Using these data, the model parameters were fitted, with a resulting acceptable agreement between model simulations and data (Fig. 3d). To further constrain the model parameters, we generated data for experiments where oligomycin was added prior to Iso stimulation (Fig. 3e). The model could reproduce also these data (Fig. 3e, lines), even though the model simulation reaches higher values than corresponding data for the Iso stimulation. The reason for these higher values is that the same model (with the same parameters) was fitted to all data simultaneously, and since the Iso stimulation in Fig. 3d reaches higher values with a lower uncertainty than the Iso stimulation in Fig. 3e, the former data weight more in the parameter estimation.

**Fig. 4:**
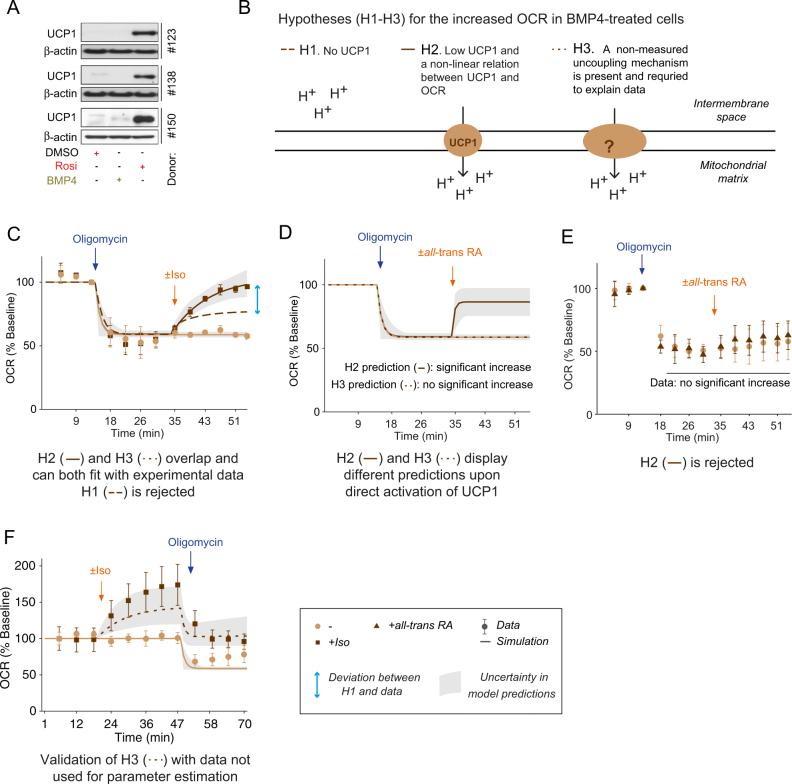
Uncoupled respiration in BMP4-derived beige adipocytes is independent of UCP1.** a** Representative Western-blot analysis (*n* = 3 subjects) of UCP1 and β-actin (loading control) in DMSO-treated, BMP4-treated and Rosi-treated cells. **b** Schematic representation of tested hypotheses (H1-H3) for increased uncoupled OCR in BMP4-derived beige adipocytes. **c** Representative dynamic OCR data (markers) and model simulation (lines) for the tested hypotheses of the increase in OCR in BMP4-treated cells under basal condition, following the addition of oligomycin and DMSO or Isoproterenol (Iso). **d**–**e** Model predictions for H2 and H3 **d** and corresponding data **e** of uncoupled (oligomycin-inhibited) OCR upon all-*trans* RA stimulation. **f** Model prediction using the final model H3, and corresponding validation data of Iso-stimulated and oligomycin-inhibited OCR. Data in panels **c**,** e** and **f** are from BMP4-treated cells, represented as mean ± SEM. *OCR* oxygen consumption rate, *BMP4* bone morphogenic protein 4, *Iso* isoproterenol, all-*trans* *RA* all-*trans* retinoic acid. See also Supplementary Figs. S5, S6

Seahorse analysis also demonstrated that all-*trans* RA increased uncoupled OCR during oligomycin-inhibition (Fig. 3f). These data were used to parametrize the model together with earlier data sets with a resulting good fit (Fig. 3f, lines). The increase in OCR upon all-*trans* RA treatment was in the same order of magnitude as the indirect activation of UCP1 (Fig. 3e). In line with these results, UCP1 depletion substantially decreased uncoupled OCR in Rosi-treated cells (Supplementary Fig. S3; cf data in Fig. 4b in ^34^). Altogether, this analysis demonstrates that the presence of UCP1 in hASCs-derived beige adipocytes correlates with an increased capacity for uncoupled respiration. Furthermore, activated UCP1 contributes substantially to the increased uncoupling observed in Rosi-treated cells.

### Uncoupled respiration in BMP4 white fat-derived beige adipocytes is UCP1 independent

As reported herein, BMP4 induces a beige adipocyte-like phenotype with high levels of mitochondrial protein, increased OCR, and a beige expression profile when compared to control white cells (Fig. 2). However, UCP1 protein is no or hardly detectable in the cells (Figs. 2e and 4a). This intriguing observation can potentially be explained with three different hypotheses of uncoupling scenarios (Fig. 4b). The first hypothesis (H1) assumes that neither UCP1 nor other uncoupling proteins/mechanisms are present in BMP4-treated cells and that the increased substrate availability in the Iso-stimulated experiments is enough to explain the increased OCR. The second hypothesis (H2) assumes that there is a non-linear relation between UCP1 protein content and OCR. H2 also assumes that there is a small amount of UCP1 protein (below detection limit) available in BMP4-treated cells and that this amount is enough to explain the increased OCR. The third hypothesis (H3) assumes that another uncoupling mechanism is needed to explain the increased OCR in BMP4-treated cells. To test these hypotheses, we used the model developed for control white and Rosi-treated cells (Fig. 3c), and implemented the hypotheses as changes in the specific beige model parameters (BA1-BA4 in Supplementary Fig. S1 and Supplemental Experimental Procedures). To handle data uncertainty in the hypothesis testing, all parameters with acceptable agreement with data according to *χ* ^2^ statistics^38^ were evaluated and visualized as gray areas (Fig. 4c, d, f and Supplementary Figs. S3–S5).

We generated dynamic OCR data for BMP4-treatment with oligomycin-inhibition and Iso-stimulation (and control) (Fig. 4c). These data, together with all gathered data from control white and Rosi-treated cells (Supplementary Fig. S4), were used to challenge and discriminate between the hypotheses. Parameters for basic mitochondrial functions were assumed the same regardless of treatment. The resulting model simulations revealed that H1 cannot fit the dynamic data and had to be rejected (Fig. 4c, dashed lines). The simulations also revealed that H2 and H3 can fit with the data (Fig. 4c, overlapping lines), i.e., that uncoupling can be present in the form of UCP1 protein at low levels, of other uncoupling mechanisms, or both.

The remaining hypotheses, H2 and H3, cannot be discriminated based on these data sets. Instead, the difference between the hypotheses is the level of UCP1 protein: H2 assumes low levels of UCP1 protein (with high activity) and H3 assumes total absence of UCP1 protein. Simulations of direct activation of UCP1 therefore gives different predictions for H2 (Fig. 4d, lines) and H3 (Fig. 4d, dotted lines) and thus a possibility to discriminate between the hypotheses. We performed the corresponding experiment using direct activation of UCP1 (all-*trans* RA), with no resulting increase in OCR (Fig. 4e). In addition, we tested if the sensitivity of UCP1 to all-*trans* RA was altered in BMP4-treated cells. To test this, we performed UCP1 rescue experiment with UCP1-encoding modified RNA. We found that experimentally induced UCP1 protein expression in BMP4-treated cells could led to UCP1 activation by all-*trans* RA and therefore all-*trans* RA can activate UCP1—if present—in BMP4-treated adipocytes. (Supplementary Fig. S6). The rejection of H2 was also confirmed in another way: using re-estimating of the model parameters with both the all-*trans* RA data (Fig. 4e) and earlier data sets (Fig. 4c and Supplementary Fig. S4). No acceptable agreement with this combined data set was found for H2 (Supplementary Fig. S5A). In summary, this means that UCP1 protein is not an active uncoupler in BMP4-treated cells. Accordingly, proton leak and cRCR were significantly changed in BMP4-treated cells only after Iso (Supplementary Fig. S5B) but not upon all-*trans* RA stimulation (Supplementary Fig. S5C), further supporting our findings.

To validate the final, non-rejected model (H3), we used the model to predict an experiment not used in model fitting. We simulated the response to stimulation with Iso (and control) followed by oligomycin, i.e. the reversed order of stimulations compared to Fig. 4c. The predicted response with uncertainty is showed in Supplementary Fig. S5D. We performed the corresponding experiment in BMP4-treated cells (Supplementary Fig. S5E) and found the prediction to agree with and overlapping the experimental data (Fig. 4f). This finding supports the final model.

**Fig. 5:**
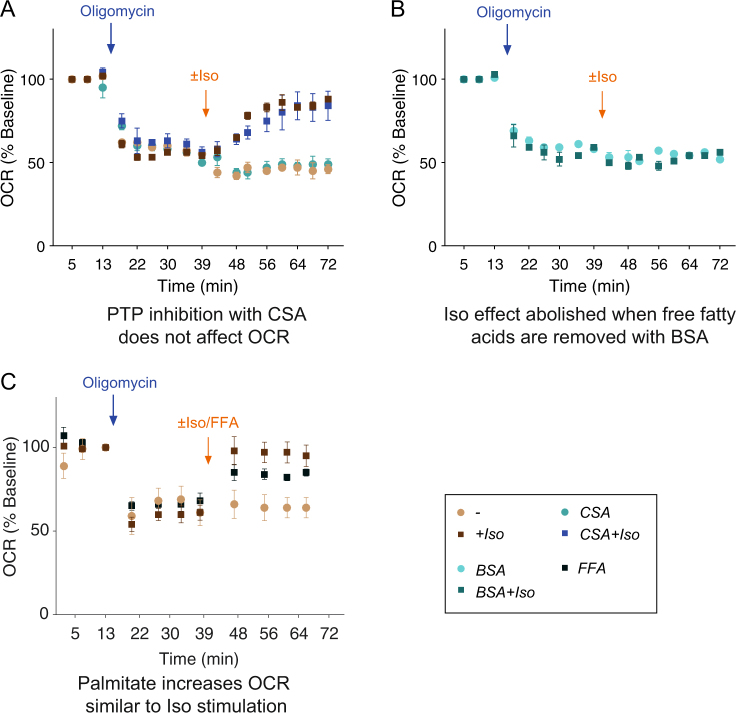
Uncoupled respiration in BMP4-derived beige adipocytes can be mimicked by addition of exogenous free fatty acids (FFA). **a**, **b** Uncoupled (oligomycin-inhibited) OCR upon Iso after inhibition of PTP. There is no difference with PTP-pore inhibition. **b** Uncoupled (oligomycin-inhibited) OCR upon Iso with scavenging of free fatty acids with BSA The Iso stimulation is completely abolished with BSA. **c** Uncoupled (oligomycin-inhibited) OCR upon exogenous addition of free fatty acid (FFA) by injection Palmitate/BSA complex. Exogenous FFA mimics the uncoupled respiration induced by Iso. Data in panels **a**–**c** are from BMP4-treated cells, represented as mean ± SEM. *OCR* oxygen consumption rate, *BMP4* bone morphogenic protein 4, *Iso* Isoproterenol, all-*trans* RA all-*trans* retinoic acid, *FFA* free fatty acid, i.e., Palmitate-BSA complex. See also Supplementary Fig. S7

In a next step, to further elucidate the mechanisms of uncoupling in BMP4-treated cells, we looked into two mechanisms proposed in the literature: the opening of mitochondrial PTP^13,39^ and the unspecific protonophoric action of intracellular free fatty acids (FFAs).^40,41^ To determine whether the mitochondrial uncoupling observed in BMP4-treated cells could be mediated by opening of the PTP, we pre-treated BMP4 white fat-derived beige adipocytes with the specific PTP inhibitor cyclosporine A (CSA) and monitored mitochondrial uncoupling. We found that the mitochondrial uncoupling observed in BMP4-treated cells is independent of PTP activity (Fig. 5a). In addition, Rosi-treated cells were not affected by the PTP-inhibitor CSA (Supplementary Fig. S7A). These results are in contrast to earlier findings in mouse brown adipocytes from UCP1-KO mice^39^ and human white adipocytes^13^ that both found a partial role for the PTP pore in Iso-induced OCR.

Control over the intracellular fatty-acid levels is essential for the correct analysis of thermogenic function in beige adipocytes and allows for specific measurement of UCP1-mediated leak respiration.^39^ To test the need for free fatty acids in BMP4-treated cells, we scavenged the extracellular free fatty acids with 2% bovine serum albumin (BSA) while performing the oligomycin + Iso test. Under this condition, uncoupled respiration was completely abolished (Fig. 5b). Rosi-treated cells, on the other hand, displayed a partial response under conditions of scavenged fatty acids (Supplementary Fig. S7B). This means that Rosi-treated cells display uncoupled respiration also without extracellular free fatty acids present, probably via UCP1. To further explore whether the released FFAs were responsible for unspecific mitochondrial uncoupling in BMP4-tretaed cells, we mimicked lipolysis by exogenous addition of palmitate-BSA complex (0.2 mM) to the assay media. Exogenous fatty acids induced leak respiration independent of UCP1 to a similar extent as treatment with Iso (Fig. 5c). Together, these data show that high levels of free fatty acids are needed for uncoupling to occur in BMP4-treated cells. However, the exact mechanisms of the uncoupling in BMP4-treated cells remains to be elucidated.

The systematic approach used herein, with combinations of several high-quality data sets, formal testing of hypotheses, and informed experimental design, demonstrate that stimulated uncoupled respiration in BMP4-treated white fat-derived beige adipocytes is independent of UCP1.

## Discussion

The results in this paper call into question the very definition of beige adipocytes. Although clarity is present when defining brown adipocytes—as cells that upon stimulation owes their thermogenic capacity due to the presence of UCP1—the definition of beige/brite adipocytes remains controversial in the field.^23,42,43^ Common definitions have relied on one or a few of the conventional biomarkers: gene expression of UCP1 and other brown-specific genes (Fig. 2b), fractions between values in an OCR analysis (Fig. 2c, d), and morphological characterizations like lipid droplet formation (Fig. 2a). All these biomarkers point towards both BMP4-treatment and Rosi-treatment giving beige adipocytes (Fig. 2). Nevertheless, the perhaps most central of all biomarkers, which surprisingly seldom is measured^24^—UCP1 protein levels—is below detection levels for the BMP4-treated cells (Fig. 2e). This means that the increased mRNA levels in this case do not lead to increased protein levels. In itself this is not surprising since systems-wide studies have shown that only one-third of the variation of protein levels are explained by mRNA levels.^44^ However, in combination with our functional observations on the beige phenotype of BMP4-treated cells, it is an intriguing observation. A similar discrepancy in UCP1 mRNA and UCP1 protein levels (without further functional characterization) have been observed in obesity,^45^ where Rosi-treated cells from both lean and obese individuals induced high levels of UCP1 mRNA, and only cells from lean individuals acquired detectable levels of UCP1 protein. Our findings of a difference between mRNA and protein levels of UCP1 can also be related to recent findings in mice, where UCP1 negative adipocytes are found that both express UCP1 mRNA and leak protons.^15^ The UCP1 negative adipocytes coexist with UCP1 positive adipocytes and are found to be in majority in at least one location (epidymal fat deposits).

Herein, we have developed a new systems biology framework based on both functional characterization and UCP1 mRNA and protein measurements in human beige cells suitable for a more comprehensive hypothesis testing (Fig. 1). This framework relies on a fit-for-purpose mathematical model (Fig. 3c), which first was shown to be able to describe the data from the Rosi-induced cells (Fig. 3d–f), and then was applied to the BMP4 data (Fig. 4). In a series of steps, this framework could devise conclusive evidence that UCP1 cannot be the uncoupling agent, but that a complementary uncoupling mechanism must be present in the BMP4-treated cells. This means that we need to update our definition of what it means to be a beige adipocyte, into one that involves a more comprehensive characterization.

Such a comprehensive characterization can be done using our new systems biology framework, which has several important strengths compared to existing options. First, our developed mathematical model is the first, to our knowledge, devoted to interpreting whole-cell Seahorse OCR data. Second, our model can consider the entire time series (in e.g., Fig. 3d) of OCR measurements and not only the conventional summary parameters (Fig. 2c, d). Finally, our analysis provides several insights that are hard to see through without the use of modeling: (a) The original data alone (Figs. 2–3, Fig. 4c) could not conclude weather UCP1 could be the uncoupling mechanism in the BMP4-treated cells: H2 remained non-rejected using these data (Fig. 4c); (b) Using an advanced uncertainty analysis,^38^ not possible to do without modeling, we could device an experiment (Fig. 4d) that was guaranteed to distinguish between the two remaining hypotheses H2 and H3: at the most one of them could have provided a correct prediction. Note that these rejections are final, meaning that H1 and H2 can be ignored from all future analyses searching for the true mechanism for the uncoupling.

It is interesting to put our observation of an UCP1-independent uncoupling mechanism into perspective of related findings. First, it has not been previously demonstrated—in human beige cells—that a pharmacologically induced uncoupler can give a beige phenotype independent of UCP1. Second, there are several indications that genetically changed mice can exhibit UCP1-independent non-shivering thermogenesis: (i) UCP1 null mice gradually exposed to cold do manage to adapt their thermogenesis,^10,46^ and (ii) adrenergic and peptide stimulation show that the thermogenic response of wild-type and UCP1-knockout mice are comparable.^11,47^ Third, it is unclear what is the exact nature of these observations of UCP1-independent thermogenesis, but several suggestions have been made: Glycerol-3-phosphate shuttle,^48^ creatine-driven substrate cycle,^14,15^ N-acyl amino acids,^16^ lipid turnover,^49^ and fatty-acid mediated uncoupling of the mitochondrial membrane.^39^ Finally, while these mechanisms in mice are currently being characterized, their precise regulation in human beige adipose cells has yet to be elucidated.

The main conclusion in this paper is that we have identified a new beige phenotype, which increased uncoupling is dominated by something else than UCP1. This conclusion could in principle be drawn already from the data in Fig. 2, which indicates increased uncoupling (Fig. 2c) with non-detectable UCP1 protein levels (Fig. 2e). However, as we have shown with an iterative modeling/experimental approach, there are alternative hypotheses that cannot be rejected based on that data alone. In addition, the main conclusion is new and controversial, and therefore needs to be thoroughly examined before it can be believed. More specifically, the non-detectable UCP1 protein levels (Fig. 2e) could e.g. simply be due to a failed experiment, or to a post-translational modification of the UCP1 protein, which still makes the protein functional, but makes it invisible to the antibody used for detection. Similarly, it could be that the low protein levels are true, but that they are sufficient to maintain function (Fig. 4b; H2). Other possible alternative hypotheses has to do with the interpretation of the time-resolved OCR data. There are several examples where time-resolved data originally analyzed without models have led to one conclusion, but where a subsequent model-based analysis has identified an alternative explanation for the same data.^25,50^ Therefore, we collected a larger data set and developed a suitable mathematical model to analyze it. We developed a new model even though there are models available for mitochondrial respiration,^31–33^ since (i) these models, even though they are highly detailed, do not include all parameters and signaling pathways needed to analyze our data (e.g., UCP1 and lipolysis), (ii) these models were not developed with the purpose of interpreting time-resolved OCR data, and (iii) these models have many parameters that have not been validated for brown, beige, or white adipocytes. Furthermore, these detailed models are so over-parametrized with respect to our available data that cell-specific parameters cannot be inferred, and that the models cannot be used to identify the key ingredient in conclusive experiment design (Fig. 1b): predictions with well-defined uncertainty boundaries (Fig. 4c, d, gray areas). For these reasons, we could not just draw the obtained conclusion from the original data (Fig. 2), but had to perform an iterative systems biology analysis (Figs. 3, 4), where new targeted experiments were designed, and where data were interpreted, using a new fit-for-purpose mathematical model.

The perhaps most important consequence of our finding is that current screens for new drugs that induce browning can be widened to also search for the induction of new mechanisms, independent of UCP1. However, if this is done, care must be taken to understand the site(s) of such uncoupled respiration to avoid undesired side effects. For instance, one of the benefits of UCP1-induced uncoupling is that it has an in-built shut-down mechanism,^51^ which other leakage mechanisms may lack. Similarly, while an increased browning of WAT sometimes may be beneficial, a similar increased leakage in other organs might be detrimental. It may therefore be important to make this induction organ-specific. Nevertheless, if such potential side effects are correctly handled, this new mechanism for increased uncoupling may turn out to be the mechanism used to at last find a treatment for one of our costliest and deadliest diseases: obesity and the metabolic syndrome.

## Methods

### Patient consent

Samples of adipose tissues were collected from patients undergoing elective surgery at Sahlgrenska University Hospital in Gothenburg, Sweden. All study subjects received written and oral information before giving written informed consent for the use of the tissue. The studies were approved by The Regional Ethical Review Board in Gothenburg, Sweden. Clinical data of subjects used in this study are reported in Supplementary Table S1.

### Isolation and culture of hASCs

Human subcutaneous WAT was obtained from healthy women undergoing elective fat removal. We isolated hASCs from the stromal vascular fraction as described earlier.^34^ hASCs were cultured in a growth medium DMEM/Ham’s F12 with 10%FBS, 10 mM HEPES, 33 µM biotin (Sigma), 17 µM pantothenate (Sigma),1 nM fibroblast growth factor (Sigma), 50 U/ml penicillin and 50 µg/ml streptomycin at 37 °C, 5% CO_2_ in air with 80% humidity. For adipocyte differentiation, 90% confluent cells were treated with DMEM/F12 with 3% FCS (PAA, Gold) supplemented with 100 nM dexamethasone (Sigma), 500 µM 3-isobutyl-1-methyxanthine (Sigma), 0.85 µM insulin, 5 nM triiodothyronine (T3, Sigma). To promote beige adipogenesis, 100 nM rosiglitazone (Rosi) or 50 ng/ml recombinant human BMP4 were included in the differentiation medium. Media was changed every other day during proliferation and differentiation, until fully differentiated (day 32). At this point, both Rosi and BMP4 were removed from the differentiation media and cells were kept in maintenance media (DMEM/F12 with 3% FCS, 100 nM dexamethasone, 0.85 µM insulin) over-night till seahorse analysis.

### Immunoblotting

Cells were lysed in modified RIPA buffer (20 mM Hepes pH7.9, 5 mM MgCl2, 25% Glycerol, 1% NP40, 150 mM NaCl, 1× PhosphoSTOP and 1× Complete mini [Roche]). Protein concentration was determined using a BCA kit (Pierce). After electrophoresis, proteins were transferred to nitrocellulose membrane for incubation with primary antibody (OXPHOS complexes (MS-604, Mitosciences), PGC1 (sc-13067, Santa Cruz), UCP1 (MAb6158, R&D), Pparγ (sc-7196, Santa Cruz), and β-actin (A5441, Sigma)).

### RNA extraction and quantitative real-time PCR (qPCR)

Total RNA was extracted using RNAeasy (Qiagen) and reversed-transcribed using the High Capacity cDNA Reverse Transcription Kit (Applied Biosystems). qPCR was performed using TaqMan Gene Expression Assays (Supplementary Table S2). TBP served as loading controls.

### XF24 Oxygen consumption analysis

OCR was measured using the XF24 Analyzer (Seahorse Bioscience). Chemicals used: Rotenone (8875, Sigma), Antimycin A (8674, Sigma), Oligomycin (O4876, Sigma), 2,4 DNP (D199303, Sigma), Isoproterenol (I6504, Sigma), ATRA (R2625, Sigma), Palmitate (P9767, Sigma), and BSA free fatty acid (A6003, Sigma). After the XF experiments cells were fixed, stained with a nuclei dye and counted to allow for normalization of oxygen consumption rates to cell number.

### Statistical analysis

Statistical analyses for qPCR and OCR experiments were performed by one-way ANOVA with Bonferroni post-test to compare all conditions or by paired or unpaired Students’ *t*-test using Prism Graph Pad.

### Software and code

The toolbox IQM Tools Pro SB (IntiQuan; http://www.intiquan.com) was used together with Matlab R2016a (MathWorks; http://mathworks.com/) in the mathematical modeling. Files to simulate the models for the hypotheses H1-H3 are found at https://github.com/elinnyman/uncoupling-beyond-UCP1.

### Data availability

Data used in the model based analysis (Figs. 3 and 4) are found at https://github.com/elinnyman/uncoupling-beyond-UCP1 together with code to reproduce the figures. These data and all other data is also available upon request.

## Electronic supplementary material

Supplemental Material

## References

[1] Yoneshiro T (2013). Recruited brown adipose tissue as an antiobesity agent in humans. J. Clin. Invest..

[2] Barbatelli G (2010). The emergence of cold-induced brown adipocytes in mouse white fat depots is determined predominantly by white to brown adipocyte transdifferentiation. Am. J. Physiol. Endocrinol. Metab..

[3] Petrovic N, Shabalina IG, Timmons JA, Cannon B, Nedergaard J (2008). Thermogenically competent nonadrenergic recruitment in brown preadipocytes by a PPARgamma agonist. Am. J. Physiol. Endocrinol. Metab..

[4] Petrovic N (2010). Chronic peroxisome proliferator-activated receptor gamma (PPARgamma) activation of epididymally derived white adipocyte cultures reveals a population of thermogenically competent, UCP1-containing adipocytes molecularly distinct from classic brown adipocytes. J. Biol. Chem..

[5] Vegiopoulos A (2010). Cyclooxygenase-2 controls energy homeostasis in mice by de novo recruitment of brown adipocytes. Science.

[6] Lee JY (2012). Triiodothyronine induces UCP-1 expression and mitochondrial biogenesis in human adipocytes. Am. J. Physiol. Cell Physiol..

[7] Schulz TJ (2011). Identification of inducible brown adipocyte progenitors residing in skeletal muscle and white fat. Proc. Natl. Acad. Sci. USA.

[8] Bordicchia M (2012). Cardiac natriuretic peptides act via p38 MAPK to induce the brown fat thermogenic program in mouse and human adipocytes. J. Clin. Invest..

[9] Shabalina IG (2013). UCP1 in brite/beige adipose tissue mitochondria is functionally thermogenic. Cell Rep..

[10] Ukropec J, Anunciado RP, Ravussin Y, Hulver MW, Kozak LP (2006). UCP1-independent thermogenesis in white adipose tissue of cold-acclimated Ucp1-/- mice. J. Biol. Chem..

[11] Granneman JG, Burnazi M, Zhu Z, Schwamb LA (2003). White adipose tissue contributes to UCP1-independent thermogenesis. Am. J. Physiol. Endocrinol. Metab..

[12] Shabalina IG, Kramarova TV, Nedergaard J, Cannon B (2006). Carboxyatractyloside effects on brown-fat mitochondria imply that the adenine nucleotide translocator isoforms ANT1 and ANT2 may be responsible for basal and fatty-acid-induced uncoupling respectively. Biochem. J..

[13] Yehuda-Shnaidman E, Buehrer B, Pi J, Kumar N, Collins S (2010). Acute stimulation of white adipocyte respiration by PKA-induced lipolysis. Diabetes.

[14] Kazak L (2015). A creatine-driven substrate cycle enhances energy expenditure and thermogenesis in beige fat. Cell.

[15] Bertholet AM (2017). Mitochondrial patch clamp of Beige adipocytes reveals UCP1-positive and UCP1-negative cells both exhibiting futile creatine cycling. Cell Metab..

[16] Long JZ (2016). The secreted enzyme PM20D1 regulates lipidated amino Acid uncouplers of mitochondria. Cell.

[17] Qian SW (2013). BMP4-mediated brown fat-like changes in white adipose tissue alter glucose and energy homeostasis. Proc. Natl. Acad. Sci. USA.

[18] Xue R (2014). Role of bone morphogenetic protein 4 in the differentiation of brown fat-like adipocytes. Am. J. Physiol. Endocrinol. Metab..

[19] Modica S, Wolfrum C (2013). Bone morphogenic proteins signaling in adipogenesis and energy homeostasis. Biochim. Biophys. Acta.

[20] Modica, S. et al. Bmp4 promotes a brown to white-like adipocyte shift. *Cell Rep.* **16**, 2243–2258 (2016). 10.1016/j.celrep.2016.07.048 27524617

[21] Elsen M (2014). BMP4 and BMP7 induce the white-to-brown transition of primary human adipose stem cells. Am. J. Physiol. Cell Physiol..

[22] Gustafson B (2015). BMP4 and BMP antagonists regulate human white and beige adipogenesis. Diabetes.

[23] Wu J, Cohen P, Spiegelman BM (2013). Adaptive thermogenesis in adipocytes: is beige the new brown?. Genes Dev..

[24] Nedergaard J, Cannon B (2013). UCP1 mRNA does not produce heat. Biochim. Biophys. Acta.

[25] Nyman E (2015). Mathematical modeling improves EC50 estimations from classical dose-response curves. FEBS J..

[26] Jullesson D, Johansson R, Rajan MR, Stralfors P, Cedersund G (2015). Dominant negative inhibition data should be analyzed using mathematical modeling--re-interpreting data from insulin signaling. FEBS J..

[27] Spalding KL (2008). Dynamics of fat cell turnover in humans. Nature.

[28] Brännmark C (2013). Insulin signaling in type 2 diabetes: experimental and modeling analyses reveal mechanisms of insulin resistance in human adipocytes. J. Biol. Chem..

[29] Nyman E (2014). A single mechanism can explain network-wide insulin resistance in adipocytes from obese patients with type 2 diabetes. J. Biol. Chem..

[30] Rajan, M. R., Nyman, E., Kjolhede, P., Cedersund, G. & Stralfors, P. Systems-wide experimental and modeling analysis of insulin signaling through FOXO1 in human adipocytes, normally and in type 2 diabetes. *J. Biol. Chem*. **291**, 15806–15819 (2016). 10.1074/jbc.M116.715763 PMC4957062 27226562

[31] Beard DA (2005). A biophysical model of the mitochondrial respiratory system and oxidative phosphorylation. PLoS. Comput. Biol..

[32] Wu F, Yang F, Vinnakota KC, Beard DA (2007). Computer modeling of mitochondrial tricarboxylic acid cycle, oxidative phosphorylation, metabolite transport, and electrophysiology. J. Biol. Chem..

[33] Vinnakota KC, Bazil JN, Van den Bergh F, Wiseman RW, Beard DA (2016). Feedback regulation and time hierarchy of oxidative phosphorylation in cardiac mitochondria. Biophys. J..

[34] Bartesaghi S (2015). Thermogenic activity of UCP1 in human white fat-derived beige adipocytes. Mol. Endocrinol..

[35] Cannon B, Nedergaard J (2004). Brown adipose tissue: function and physiological significance. Physiol. Rev..

[36] Rial E (1999). Retinoids activate proton transport by the uncoupling proteins UCP1 and UCP2. EMBO J..

[37] Tomas P (2004). Activation by retinoids of the uncoupling protein UCP1. Biochim. Biophys. Acta.

[38] Cedersund G (2012). Conclusions via unique predictions obtained despite unidentifiability--new definitions and a general method. FEBS J..

[39] Li Y, Fromme T, Schweizer S, Schottl T, Klingenspor M (2014). Taking control over intracellular fatty acid levels is essential for the analysis of thermogenic function in cultured primary brown and brite/beige adipocytes. EMBO Rep..

[40] Di Paola M, Lorusso M (2006). Interaction of free fatty acids with mitochondria: coupling, uncoupling and permeability transition. Biochim. Biophys. Acta.

[41] Wojtczak L, Schonfeld P (1993). Effect of fatty acids on energy coupling processes in mitochondria. Biochim. Biophys. Acta.

[42] Rosenwald M, Wolfrum C (2014). The origin and definition of brite versus white and classical brown adipocytes. Adipocyte.

[43] Harms M, Seale P (2013). Brown and beige fat: development, function and therapeutic potential. Nat. Med..

[44] Vogel C (2010). Sequence signatures and mRNA concentration can explain two-thirds of protein abundance variation in a human cell line. Mol. Syst. Biol..

[45] Carey AL (2014). Reduced UCP-1 content in in vitro differentiated beige/brite adipocytes derived from preadipocytes of human subcutaneous white adipose tissues in obesity. PLoS One.

[46] Meyer CW (2010). Adaptive thermogenesis and thermal conductance in wild-type and UCP1-KO mice. Am. J. Physiol. Regul. Integr. Comp. Physiol..

[47] Veniant MM (2015). Pharmacologic effects of FGF21 are independent of the “browning” of white adipose tissue. Cell Metab..

[48] Flachs P (2011). Synergistic induction of lipid catabolism and anti-inflammatory lipids in white fat of dietary obese mice in response to calorie restriction and n-3 fatty acids. Diabetologia.

[49] Grimpo K (2014). Brown adipose tissue dynamics in wild-type and UCP1-knockout mice: in vivo insights with magnetic resonance. J. Lipid Res..

[50] Almquist J (2016). Unraveling the pharmacokinetic interaction of ticagrelor and MEDI2452 (Ticagrelor antidote) by mathematical modeling. CPT Pharmacometrics Syst. Pharmacol..

[51] Divakaruni AS, Brand MD (2011). The regulation and physiology of mitochondrial proton leak. Physiology.

