# Supplementary material for: Systems biology reveals uncoupling beyond UCP1 in human white fat-derived beige adipocytes
Source: NPJ Syst Biol Appl. 2017 Oct 3;3:29. doi: 10.1038/s41540-017-0027-y (PMC5626775; doi:10.1038/s41540-017-0027-y)
Supplement: Supplementary file 1 — Supplemental Material [file 41540_2017_27_MOESM1_ESM.pdf]

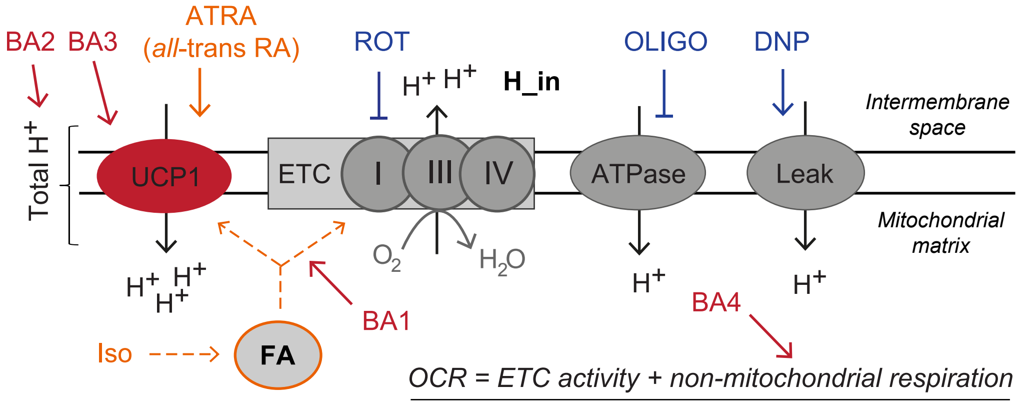

$$\frac{d}{dt}(\mathbf{H\_in}) = \mathrm{ETC}(\mathbf{H\_in},\mathbf{FA})-(\mathrm{LEAK}+\mathrm{UCP1}(\mathbf{FA})+\mathrm{ATPase})\cdot\mathbf{H\_in}$$

$$\frac{d}{dt}(\mathbf{FA}) = k\_bAR\cdot Iso - k\_1\cdot\mathbf{FA}$$

$$\frac{d}{dt}(OLIGO) = k\_oligo\cdot(oligo-OLIGO)$$

$$\frac{d}{dt}(ATRA) = k\_atra1\cdot(k\_atra2\cdot atra-ATRA)$$

$$\mathrm{ETC}(\mathbf{H\_in},\mathbf{FA}) = (k\_fa1\cdot\mathbf{FA}\cdot(1+BA1) + k\_H\cdot(k\_totH\cdot(1+BA2)-\mathbf{H\_in}))\cdot(1-ROT)$$

$$\mathrm{UCP1}(\mathbf{FA}) = (k\_ucp1+k\_fa2\cdot\mathbf{FA})\cdot BA3\cdot(ATRA+1)$$

$$\mathrm{LEAK} = k\_leak+DNP$$

$$\mathrm{ATPase} = k\_atp\cdot(1-OLIGO)$$

$$\mathrm{OCR} = \mathrm{ETC}(\mathbf{H\_in},\mathbf{FA})+k\_nonmit\cdot(1+BA4)$$

Figure S1, related to Figure 3: The developed mathematical model for oxygen consumtion in adipocytes. Here the color coding helps to understand how the mathematical model is built up with ordinary differential equations. The main states of the model: protons in the intermembrane space (H_in) and fatty acids (FA) are indicated in bold. Browning agent effects (BA1-BA4) are shown in red, the input signals all-trans retionic acid (ATRA) and isoproterenol (Iso) are shown in orange, and the chemicals used in a mitochondrial stress test (rotenone, ROT; oligomycin, OLIGO; and DNP) are shown in blue. For a full description of all equations in the model, see the Supplemental Experimental Procedures.

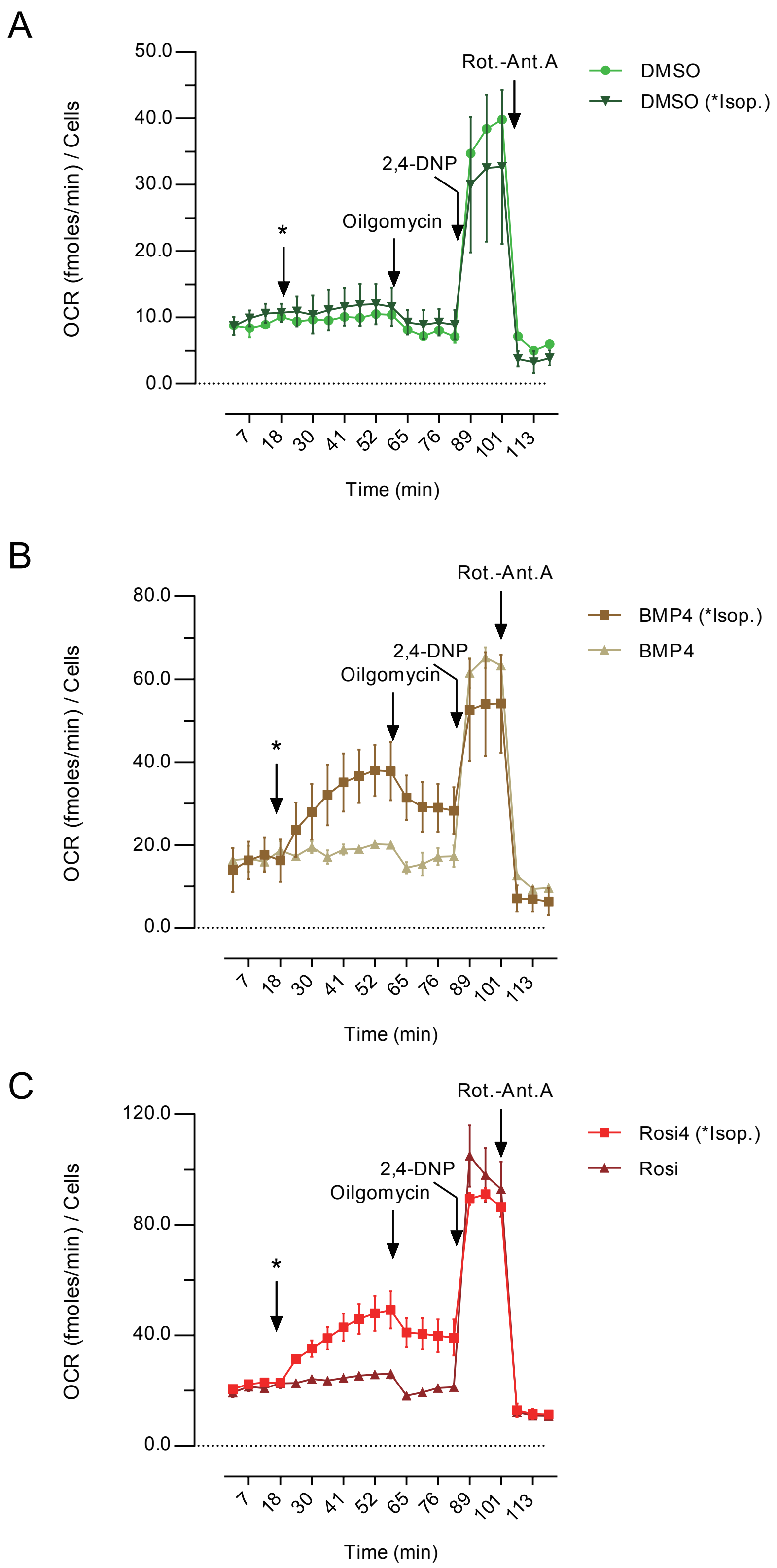

Figure S2, related to Figure 2: Mitochondrial stress test in differentiated human adipocytes
OCR in differentiated hASCs druing a mitochondrial stress test, i.e. under basal conditions, with/without addiition of Iso (*), addition of oligomycin, in the presence of 2,4DNP, and with rotenone/antimycin added.
A) Control white adipocytes differentitaed in DMSO.
B) Beige adipocytes differentiated with BMP4 added.
C) Beige adipocytes differentiated with Rosi added.

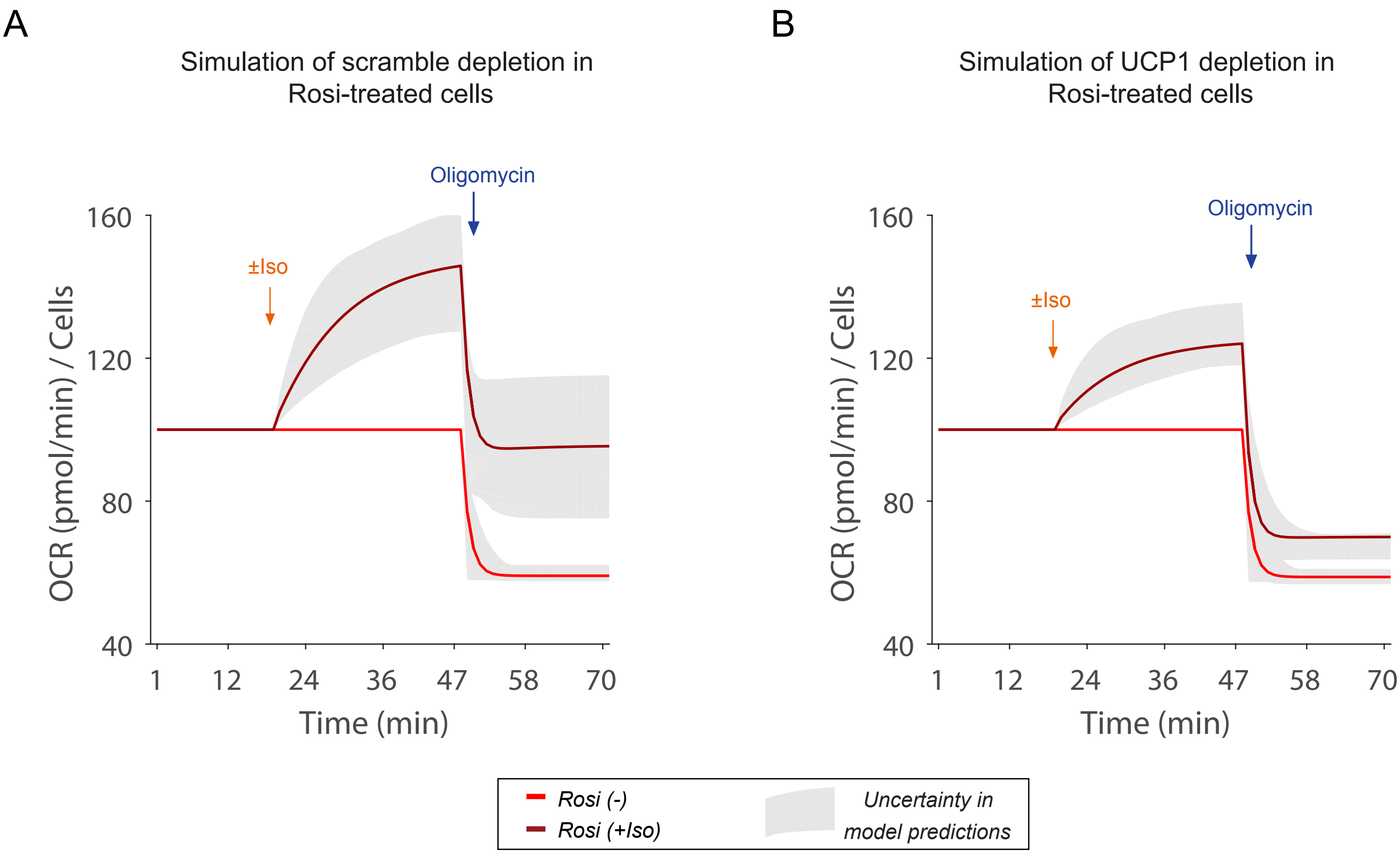

Figure S3, related to Figure 3: UCP1 depletion in beige adipocytes treated with rositaglitazone.
A) Model simulations of scramble control cells (without UCP1 depletion) with and without isoproterenol (Iso).
B) Model simulation of with UCP1 depletion with and without isoproterenol (Iso).
For a full description of the simulations of UCP1 depletion, see the Supplemental Experimental Procedures.

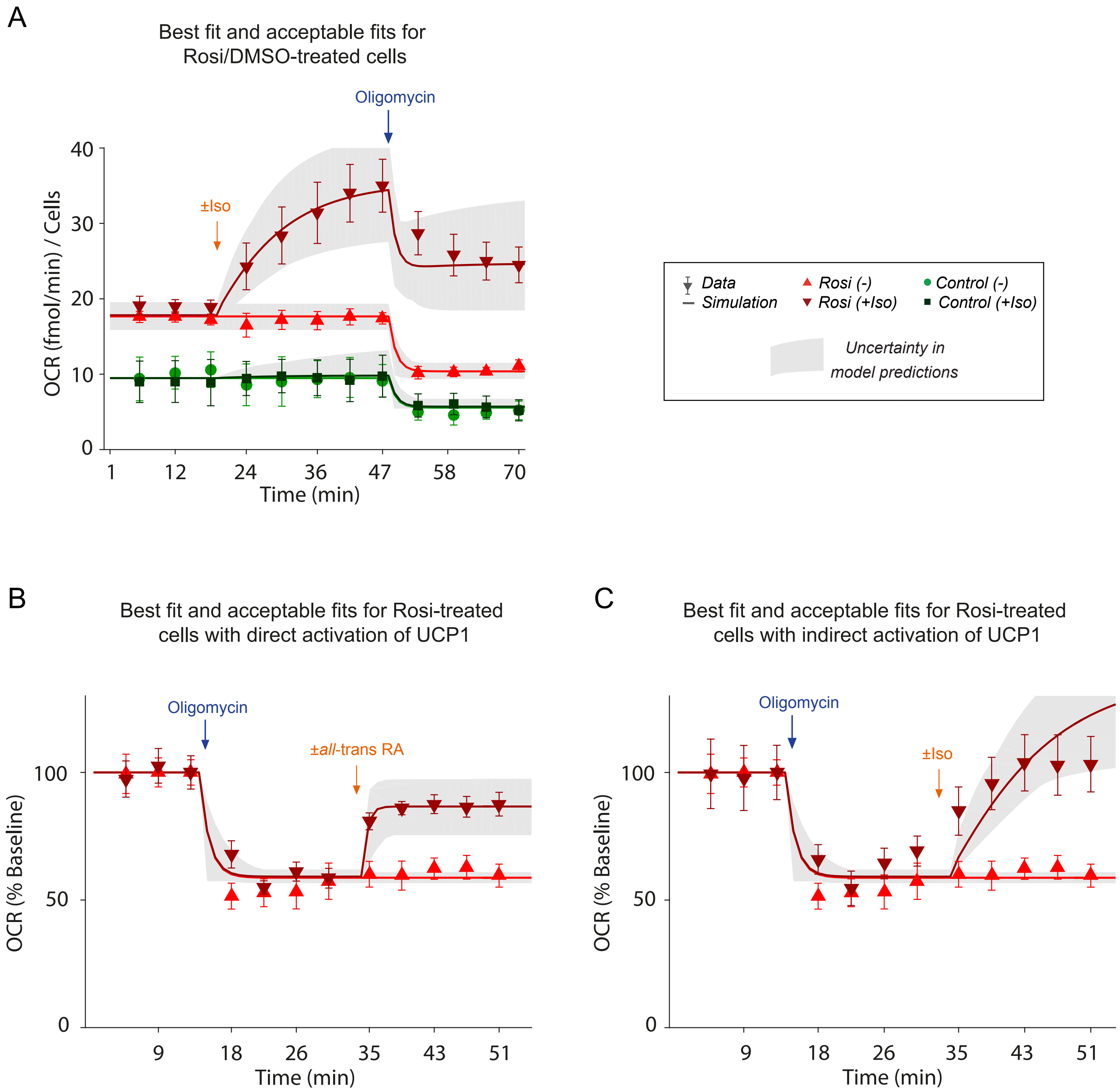

Figure S4, related to Figure 4: An approximation of the acceptable sets of parameters that fit all data.
A) Model simulations with uncertainty for Rosi-treated and control cells in a stress test with oligomycin with and without addition of isoproterenol (Iso).
B) Model simulations with uncertainty for Rosi-treated and control cells with oligomycin added to inhibit ATP production and *all*-trans retionic acid (*all*-trans RA) for a direct UCP1 activation.
C) Model simulations with uncertainty for Rosi-treated and control cells with oligomycin added to inhibit ATP production and Iso added for an indirect activation of UCP1.
For a description of data, see Figure 3, and for a full description of all equations in the model, see the Supplemental Experimental Procedures.

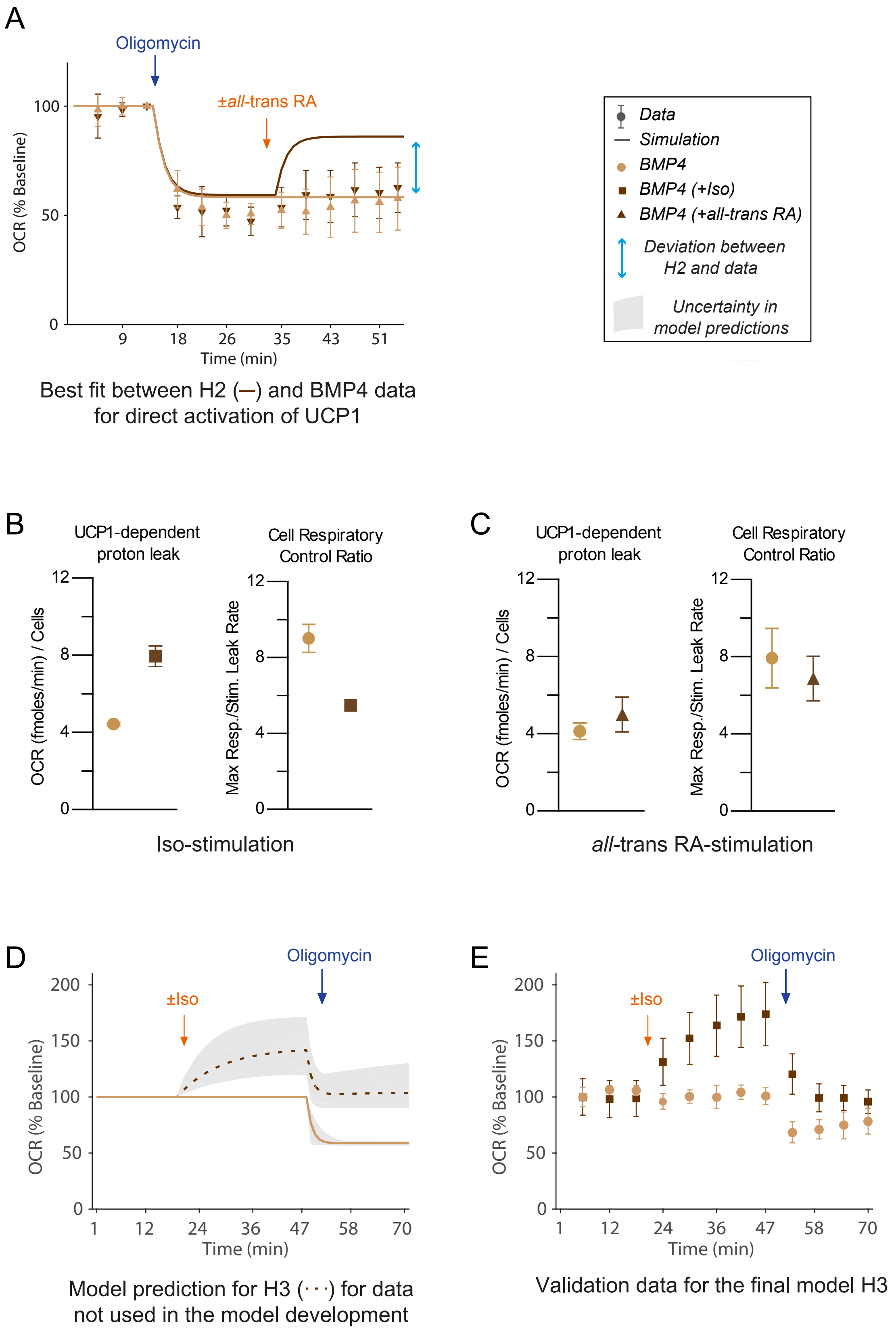

Figure S5, related to Figure 4: Rejection of hypothesis H2 and validation of hypothesis H3

A) To be sure of the rejection of hypotesis H2, all data was fitted simultaneously using the H2 model structure. It was not possible to obtain a good fit with the direct activation data with *all*-trans retionic acid (*all*-trans RA).

B) Proton leak and cell respiratory control ratio in BMP4-treated cells under basal or stimulated (Iso) conditions.

C) Proton leak and cell respiratory control ratio in BMP4-treated cells under basal or stimulated (*all*-trans RA) conditions.

D) Model simulations for H3 predict the response to Iso (or control) followed by Oligomycin in BMP4-treated cells. The grey area represent the uncertainty of the prediction.

E) Data for the experiment simulated in D used to validate the final accepted hypothesis H3.

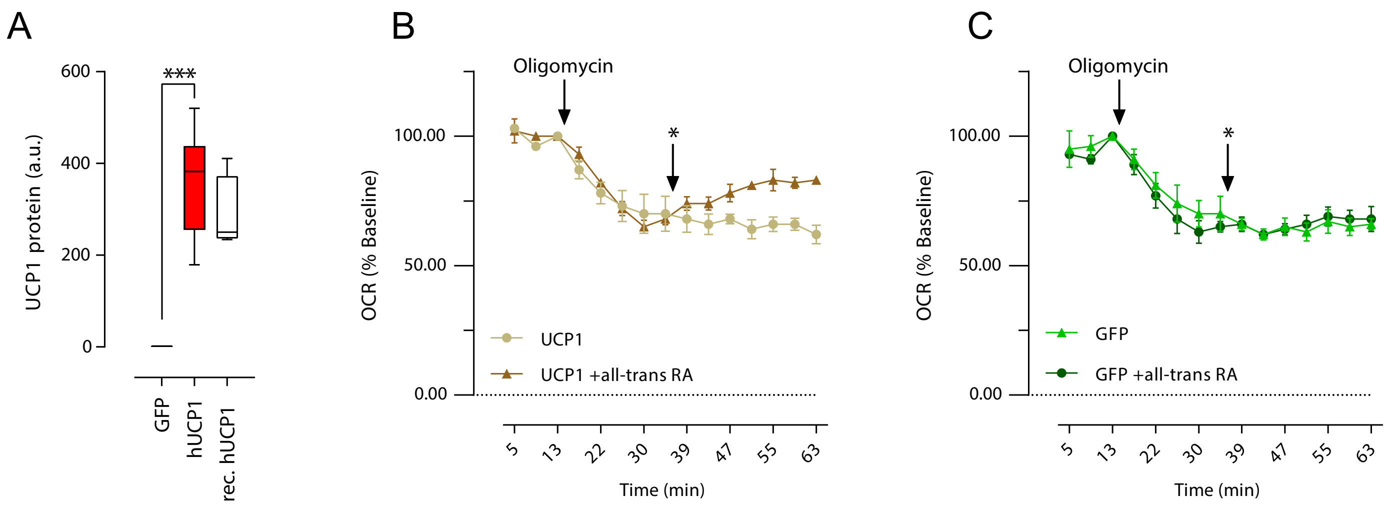

Figure S6, related to Figure 4: Experimentally expressed UCP1 protein can be activated by all-trans RA to increase OCR in BMP4 treated beige adipocytes

A) Human UCP1 levels were measured using Meso Scale Discovery (MSD, Rockville, MD, USA). GFP modified RNA has been used as scramble control, recombinant human UCP1 (rec.hUCP1) as positive control

B) Uncoupled (oligomycin-inhibited) OCR upon all-trans RA stimulation in UCP1 positive cells. UCP1 activation by all-trans RA is not affected in BMP4-treated adipocytes.

C) Uncoupled (oligomycin-inhibited) OCR upon all-trans RA stimulation in GFP positive cells. No increased in OCR reflecting no UCP1 activity.

Data in panel A-C are from BMP4 treated cells, represented as mean ± SEM. rec.hUCP1, recombinant human UCP1; OCR, oxygen consumption rate; all-trans RA, all-trans retinoic acid; GFP, green flourescent protein.

A

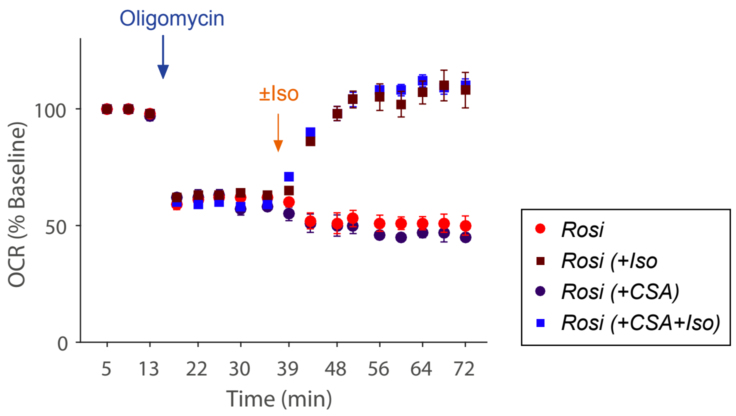

PTP inhibition does not affect OCR

B

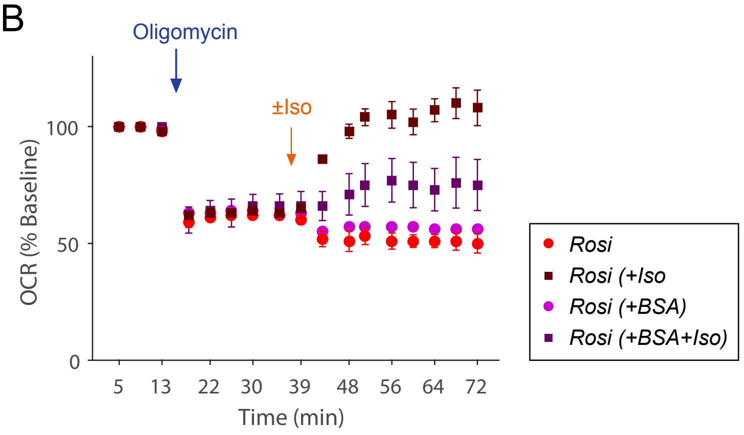

The Iso effect is reduced more than 50 %
when free fatty acids are removed

Figure S7, related to Figure 5: The PTP-pore is also not involved in uncoupling in Rosi-treated cells
A) Uncoupled (oligomycin-inhibited) OCR upon Iso-treatment w/o inhibition of PTP with CSA. There is no difference w/o PTP-pore inhibition.
B) Uncoupled (oligomycin-inhibited) OCR upon Iso w/o scavenging of free fatty acids with BSA. BSA lowers but do not completely abolish OCR.
Data in panel A-B are from Rosi-treated cells, represented as mean ± SEM. OCR, oxygen consumption rate; Iso, Isoproterenol; all-trans RA, all-trans retinoic acid; CSA, cyclosporine A; BSA, bovine serum albumin.

Table S1. Clinical data of subjects used in this study.

Subject	Gender	Age	Initial weight (kg)	Actual weight (kg)	Height (cm)	BMI	Medications/disease
**#123**	F	34	105	81	173	27	vitamins/none
**#138**	F	32	138	67	167	24	vitamins/none
**#150**	F	24	128	63	156	26	None/none

Table 2S. Taqman® Primer sequences used in qualitative RT-PCR.

Gene	Assay ID	Reference
TBP	Hs00427620_m1	NM_003194.4
UCP1	Hs00222453_m1	NM_021833.4
Prdm16	Hs00922674_m1	NM_022114.3
Cpt1b	Hs03046298_s1	NM_004377.3
AdipoQ	Hs00605917_m1	NM_001177800.1
Fabp4	Hs01086177_m1	NM_001442.2
PPARGC1A (PGC1α)	Hs01016724_m1	NM_013261.3
Cited1	Hs00366310_m1	NM_004143.3
Tmem26	Hs00415619_m1	NM_178505.6
Pparg	Hs01115513_m1	NM_005037.5
Zic1	Hs00602749_m1	NM_003412.3

Supplemental Experimental Procedures

Basic principles of mathematical modeling in biology

Mathematical modeling is a formalized way of testing mechanistic hypotheses using experimental data and prior knowledge. A mechanistic hypothesis corresponds to an idea of which mechanisms that are essential to produce the observed behavior in the experimental data. For the translation of the hypothesis to such specific models, we use ordinary differential equations (ODEs) of the following format:

$$dx/dt = f(x,p)$$
$$y = g(x,p)$$

where x represents the states, that corresponds to concentrations of substances, p the parameters, that corresponds to the kinetic rate constants, and y contains the measured signals, that corresponds to the experimental datasets. The non-linear functions f and g describe a set of specific dynamic/mechanistic assumptions.

The parameters of biological models have in principle always unknown values (Nyman et al., 2012). We therefore need to define realistic lower and upper limits of their values. Using these limits, we search through a defined space of possible parameters in the search for *acceptable parameters*. Such acceptable parameters are parameters that give a good enough agreement between model simulations and experimental data. The search for acceptable parameters is an optimization problem based on the least square error:

$$V(p) = \sum_{i=1}^{N} \frac{(y(i) - \hat{y}(i,p))^2}{\sigma(i)^2}$$

where p is the tested values of the parameters, $V(p)$ is referred to as the cost function, $y(i)$ is the measurement data, $\hat{y}(i,p)$ is the simulated curve, $\sigma(i)$ is the standard error of the mean in the measurement data. The summation is over all measured data points. The agreement between model simulation and experimental data can be studied visually and/or formally tested e.g. with a χ^2-test (Cedersund and Roll, 2009). We use a χ^2-test with 95% confidence to define the set of acceptable parameters.

The outcome of the optimization process decides the next step in the hypothesis testing cycle. If the agreement between model and data is unacceptable from a statistical point of view, the model and corresponding hypothesis are rejected. The rejection is a final conclusion and therefore the next step will be to test another hypothesis. The other possible outcome from the optimization process is that the agreement between model and data is

statistically acceptable, i.e. the model cannot be rejected. This is not a final conclusion, since new datasets may not be in agreement with this so far acceptable model/hypothesis. The next step is therefore to gather all acceptable parameters, i.e. the parameters that give model simulations with a statistical agreement with data, and search for unique predictions that are shared among these parameters. These unique predictions can be used in the design of new experiments, e.g. to be able to discriminate between several acceptable models/hypotheses.

A mathematical model for oxygen consumption in beige and white adipocytes

The mathematical model for oxygen consumption in adipocytes was developed to contain only necessary components to be able to analyze the available data from beige and white adipocytes (Figures 3 and 4).

Basal model equations

The basis of the model is the flow of protons over the inner mitochondrial membrane (Figure S1). The number of protons in the intermembrane space (H_{in}) is changing with the following ODE:

$$d/dt(H_{in}) \; = \; ETC(H_{in}, FA) - (ATPase + LEAK + UCP1(FA)) \cdot H_{in}$$

The flow of protons is divided in four parts where the electron transport chain (ETC) pumps electrons to increase the proton gradient, and ATPase uses this proton gradient to produce ATP. The two other flows are unspecific leakage ($LEAK$) and UCP1-dependent leakage ($UCP1$), also referred to as uncoupled respiration.

The pumping of protons through ETC depends on substrate availability, for example fatty acids (FA), and this effect is potentiated by browning agents (BA1), such as rosiglitazone (Rosi) and BMP4. Several effects of browning agents are allowed in the model (BA1-BA3), and these are handled as continuous parameters optimized for best fit with data. The effects are allowed to be different for different browning agents ($BA1_{rosi}, BA2_{rosi}$ etc). The flow of protons also depends on the existing proton gradient, which is calculated as the difference between total protons (k_{totH}) and H_{in}. The total proton number depends on added browning agents (BA2), since the total number of mitochondria increases with browning.

$$ETC(H_{in}, FA) = k_{fa1} \cdot FA \cdot (1 + BA1) + k_H \cdot (k_{totH} \cdot (1 + BA2) - H_{in})$$

The flow of protons down the gradient, used to produce ATP, is assumed to be constant.

$$ATPase = k_{atp}$$

The leak through the membrane is also assumed to be constant.

$$LEAK = k_{leak}$$

The uncoupled respiration occurs only if browning agents are added (BA3), and if UCP1 is activated by FA. BA3 is a continuous parameter optimized for best fit with data and is allowed to be different for different browning agents.

$$UCP1(FA) = (k_{ucp1} + k_{fa2} \cdot FA) \cdot BA3$$

The measured oxygen consumption rate (OCR) is the sum of ETC and non-mitochondrial respiration (k_{nonmit}), i.e. the measured respiration when ETC = 0. The non-mitochondrial respiration depends on the addition of browning agents (BA4).

$$OCR = ETC(H_{in}, FA) + k_{nonmit} \cdot (1 + BA4)$$

<u>Simulating isoproterenol and all-trans retinoic acid stimulation</u>

Two different agents are used to stimulate uncoupling: isoproterenol (Iso) that works indirectly via FA, and *all*-trans retinoic acid (ATRA) that directly binds to UCP1. Both these factors are included in the model as discrete variables that are switched from 0 to 1 when added.

The Iso effect (iso) on FA is described by a single ODE that merges several steps of intracellular signaling from beta-adrenergic receptors to fatty acid release. Two parameters (k_{bAR} and k_1) were needed for a good agreement between model simulations and data.

$$d/dt(FA) = k_{bAR} \cdot iso - k_1 \cdot FA$$

The direct effect of ATRA on UCP1 is implemented to be present only if UCP1 is present.

$$UCP1(FA) = (k_{ucp1} + k_{fa2} \cdot FA) \cdot BA3 \cdot (ATRA + 1)$$

To obtain the observed delayed dynamics for the ATRA response, an ODE for ATRA is used, where the input ($atra$) is changed from 0 to 1 when ATRA is added and k_{atra1} and k_{atra2} are optimized for best agreement with data. Two parameters were needed for good agreement with data.

$$d/dt(ATRA) = k_{atra1} \cdot (k_{atra2} \cdot atra - ATRA)$$

Simulating a mitochondrial stress test

A mitochondrial stress test measures the uncoupled respiration, the maximal respiratory capacity, and the non-mitochondrial respiration by adding agents that block ATPase, increase leakage, and block ETC. In a simulation, these agents are added to the model at the time-points given by the experimental protocol.

In the simulation of a stress test, first oligomycin (OLIGO) is applied to inhibit ATPase.

$$ATPase = k_{atp} \cdot (1 - OLIGO)$$

Because of the dynamics in data, OLIGO is not a simple parameter that is set to 1 when oligomycin is added. Instead an ODE describes the dynamics of the OLIGO inhibition, where the discrete variable $oligo$ is changed from 0 to 1 when oligomycin is added and the parameter k_{oligo} is optimized for best agreement with data. One parameter was enough for good agreement with data.

$$d/dt(OLIGO) = k_{oligo} \cdot (oligo - OLIGO)$$

In the next part of the stress test, DNP is added to increase the unspecific leakage. This value of the input parameter DNP is changed between different simulations to be able to fit with the DNP data (see below section: Model input).

$$LEAK = k_{leak} + DNP$$

In the final part of the stress test, rotenone (ROT) is added to completely shut down the ETC. The value of ROT is changed from 0 to 1 when added.

$$ETC(H_{in}, FA) = \left(k_{fa1} \cdot FA \cdot (1 + BA1) + k_H \cdot (k_{totH} \cdot (1 + BA2) - H_{in})\right) \cdot (1 - ROT)$$

With these equations, the model can simulate the experiments for additions of Iso and *all*-trans retinoic acid, as well as for the mitochondrial stress test. The simulations can be done with different values of the model parameters. The initial conditions of the model states are set to 0 for H_{in}, FA, OLIGO, and ATRA.

$$H_{in}(0) = 0$$
$$FA(0) = 0$$
$$OLIGO(0) = 0$$
$$ATRA(0) = 0$$

The model is simulated to a steady state for the given parameter values and the states are updates to new initial values before each change in the input.

Simulating UCP1 depletion

To be able to fit with the scramble experiments, i.e. the control experiments to the UCP1 depletion measurements in Rosi-treated cells (Figure S3A-B), an extra parameter was needed (k_{depl}). The extra parameter reduces the effect of Iso according to data in Figure 4B in (Bartesaghi et al., 2015). The reason for the reduced effect of Iso can be due to the transfection process.

$$d/dt(FA) = k_{bAR} \cdot iso \cdot k_{depl} - k_1 \cdot FA$$

This extra parameter was estimated for best agreement with data and used only to simulate the UCP1 depletion and scramble experiments (Figure S3A-B). To simulate UCP1 depletion, $BA3$ is set to 0.01.

The full set of model equations

$$d/dt(H_{in}) = ETC(H_{in}, FA) - (LEAK + UCP1(FA) + ATPase) \cdot H_{in}$$
$$d/dt(FA) = k_{bAR} \cdot iso \cdot k_{depl} - k_1 \cdot FA$$
$$d/dt(OLIGO) = k_{oligo} \cdot (oligo - OLIGO)$$
$$d/dt(ATRA) = k_{atra1} \cdot (k_{atra2} \cdot atra - ATRA)$$

$$H_{in}(0) = 0$$
$$FA(0) = 0$$
$$OLIGO(0) = 1$$
$$ATRA(0) = 1$$

$$ETC(H_{in}, FA) = \left(k_{fa1} \cdot FA \cdot (1 + BA1) + k_H \cdot (k_{totH} \cdot (1 + BA2) - H_{in})\right) \cdot (1 - ROT)$$
$$UCP1(FA) = \left(k_{ucp1} + k_{fa2} \cdot FA\right) \cdot BA3 \cdot (ATRA + 1)$$
$$LEAK = k_{leak} + DNP$$
$$ATPase = k_{atp} \cdot (1 - OLIGO)$$

$$OCR = ETC(H_{in}, FA) + k_{nonmit} \cdot (1 + BA4)$$

One set of acceptable model parameter values

$k_{totH} = 1.31$ | $k_{nonmit} = 1.00$ | $BA1_{rosi} = 6.18$ | $BA1_{BMP4} = 0.532$

$k_{bAR} = 1.00$ | $k_{olig} = 0.661$ | $BA2_{rosi} = 0.865$ | $BA2_{BMP4} = 2.15$

$k_1 = 0.117$ | $k_{atra1} = 0.600$ | $BA3_{rosi} = 0.957$ | $BA3_{BMP4} = 1.00$

$k_{atp} = 1.16$ | $k_{atra2} = 16.8$ | $BA4_{rosi} = BA2_{rosi}$ | $BA4_{BMP4} = BA2_{BMP4}$

$k_{FA1} = 0.45$ | $k_{FA2} = 0.0825$

$k_H = 12.7$ | $k_{depl} = 0.418$

$k_{ucp1} = 0.0534$

$k_{leak} = 0.639$

The example values for the BA-parameters show that Rosi has a higher FA-effect at ETC (BA1) than BMP4 and that and BMP4 has a higher increase in proton number and non-mitochondrial respiration (BA2) than Rosi. The BA3 effect has to do with UCP1 and different values are used for the different tested hypotheses for UCP1 (see section below: Hypothesis testing for BMP4 data).

Model input parameters

Model input parameters are changed between off/on when they are added according to the experimental protocol.

$iso = 0/1$ | $oligo = 0/1$ | $DNP = 0/(10; 5; 20)$ | $ROT = 0/1$

$atra = 0/1$

The input parameter atra is optimized to fit data. The input parameter DNP will receive different values for different experimental conditions. In white adipocytes (control cells), DNP = 10. In Rosi-treated beige adipocytes, DNP = 5 when iso is present and DNP = 20 when iso is not present. As seen in Figure 3D, the effect of DNP is lower in Rosi-treated cells in the presence of iso. The flexibility of the DNP input parameter will not affect any of the conclusions herein since the conclusions are based on data without DNP (i.e. Figure 4C-E).

The same values for the input parameters are used for both Rosi and BMP4 simulations.

Hypothesis testing for BMP4 data

Three hypotheses were tested to explain the BMP4 data: Hypothesis 1 (H1), Hypothesis 2 (H2), and Hypothesis 3 (H3) (Figure 4B). In the hypothesis testing, parameter values were estimated to be in agreement with control, Rosi, and BMP4 data simultaneously. Also, a qualitative agreement with UCP1 depletion data from Rosi-treated cells should be obtained.

H1 tested if uncoupling via UCP1 was redundant, i.e. if UCP1 could be removed ($BA3_{BMP4} = 0$) and if the other effects of BMP4 ($BA1_{BMP4}$ and $BA2_{BMP4}$) were sufficient to explain data. The expression for $UCP1$ used to test H1:

$$UCP1 = \left(k_{ucp1} + k_{fa2} \cdot FA\right) \cdot 0 \cdot (ATRA + 1) = 0$$

No parameter values could fit with all data simultaneously with this expression for $UCP1$ and H1 was rejected (Figure 4C).

H2 tested if the low detected UCP1 protein could give rise to a high level of uncoupling, and thus explain all data. In H2, the effect of BMP4 on UCP1 ($BA3_{BMP4}$) was optimized for best agreement with data and this was achieved for $BA3_{BMP4} = 1.00$. The expression for $UCP1$ used to test H1:

$$UCP1 = \left(k_{ucp1} + k_{fa2} \cdot FA\right) \cdot BA3_{BMP4} \cdot (ATRA + 1)$$

H3 tested if another uncoupling mechanism (UCM) was needed to fit with all available data. In H3, UCP1 was removed from the model and replaced with an analog equation where $BA3_{BMP4}$ was optimized for the best agreement with data which was achieved for $BA3_{BMP4} = 1.00$. The expressions for $UCP1$ and UCM used to test H3:

$$UCP1 = \left(k_{ucp1} + k_{fa2} \cdot FA\right) \cdot 0 \cdot (ATRA + 1) = 0$$
$$UCM = (k_{ucm} + k_{fa2} \cdot FA) \cdot BA3_{BMP4}$$

The parameter k_{ucm} was set to the same value as k_{ucp1} since this gave a good agreement with data.

To be able to discriminate between H2 and H3, we performed predictions for direct activation of UCP1, i.e. addition of ATRA. The predictions were made for all found acceptable parameters. To visualize the found acceptable parameters, the set of parameter vectors containing a maximal or a minimal value of any of the model parameters was chosen and simulated. From these simulations, all maximal and minimal simulated values were picked and a grey area was visualized in between these simulated values (Figure 4C-D). After collection of ATRA-data (Figure 4E), a visual inspection was enough to reject H2. We also tried to fit H2 to the ATRA-data, but no agreement was found (Figure S5A). This procedure with predictions and uncertainty of predictions is detailed in (Cedersund, 2012).

Supplemental references

Bartesaghi, S., Hallen, S., Huang, L., Svensson, P.A., Momo, R.A., Wallin, S., Carlsson, E.K., Forslow, A., Seale, P., and Peng, X.R. (2015). Thermogenic activity of UCP1 in human white fat-derived beige adipocytes. Mol Endocrinol *29*, 130-139.

Cedersund, G. (2012). Conclusions via unique predictions obtained despite unidentifiability--new definitions and a general method. FEBS J *279*, 3513-3527.

Cedersund, G., and Roll, J. (2009). Systems biology: model based evaluation and comparison of potential explanations for given biological data. FEBS J *276*, 903-922.

Nyman, E., Cedersund, G., and Stralfors, P. (2012). Insulin signaling - mathematical modeling comes of age. Trends Endocrinol Metab *23*, 107-115.
